# The dilemma of raising awareness “responsibly”: The need to discuss controversial research with the public raises a conundrum for scientists: when is the right time to start public debates?

**DOI:** 10.15252/embr.201541853

**Published:** 2016-03-17

**Authors:** Brigitte Nerlich, Carmen McLeod

**Affiliations:** ^1^ Institute for Science and SocietyUniversity of NottinghamNottinghamUK; ^2^ Centre for Biomolecular SciencesSynthetic Biology Research CentreUniversity of NottinghamNottinghamUK

**Keywords:** S&S: Media & Publishing, S&S: Politics, Policy & Law

## Abstract

Scientists are often called upon to discuss their research publicly. These discussions are harder if the research is controversial or might frighten or worry the public. When is the right time to start a public debate and who should start the debate by highlighting potential risks and benefits?

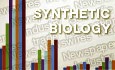

Science is often the best and sometimes the only means to address health, environmental, economic and other challenges. Debate with the public about research, its results and possible applications ensures a rational, informed process to find sustainable solutions. However, there is a conundrum at the heart of science communication, outreach activities, public or upstream engagement and Responsible Research and Innovation (RRI): When is it best to engage with the public about a controversial or emerging field of science; before or after people have become aware of the issue and begun talking about it? The timing has consequences: RRI, for example, relies on early public engagement and debate in order to guide research and applications and to ensure research and innovation align with public needs and values. However, early communication about controversial research raises the risk of creating unfounded hopes and fears. Here, we explore the contours of this conundrum by focusing on synthetic biology. Despite our focus, the issues actually affect all types of science communication and RRI activities. In some fields, such as climate change, the issues have been discussed more openly than in others. We aim to learn from these discussions when trying to answer the question: When is it the right time to responsibly raise awareness about synthetic biology?

Social scientists have analysed science communication and public engagement for many years, focusing in particular on constructions of “the public”. They have shown that publics are plural and diverse and often able to offer “lay expertise” on specific issues. This means that a simple communication model that stresses promoting public understanding, acceptance and trust in science is problematic. More recently, social scientists have begun to foster a new approach—called RRI—that encourages public engagement from the beginning of the research process, engagement that is regarded as crucial for making science more responsive to public needs. This approach has been taken, for example, in regard to the contentious issues raised or emphasized by genome editing with CRISPR. However, although many experts and commentators have appealed for a public debate, there has not yet been much practical advice about how to conduct such a debate, nor when it can or should be done responsibly.

… early communication about controversial research raises the risk of creating unfounded hopes and fears

This raises a series of questions which all relate to issues of responsibility. Is it the responsibility of social scientists or natural scientists to raise awareness of contested scientific issues? Or is this the task of professional communicators and intermediaries? And most importantly: When should this be done? Should scientists and/or intermediaries stimulate a debate about contentious issues before such a debate occurs naturally, something that is to some extent reflected in traditional and online media? Should one leave the groundwork of organizing public deliberation to natural scientists, as in the gene/genome/CRISPR debate? Who decides which issues—risks, benefits, uncertainties—should be brought to public attention and how; that is, who decides which information to foreground and which to background and how to frame these issues? Can this be achieved in a neutral way, or is bias unavoidable? What are the risks to social and natural scientists themselves when getting involved in shaping a public debate? Will they be branded “advocates” or “activists” and lose their neutrality, independence and credibility?

The issue of science advocacy is a complex topic and has led to heated discussions among scientists, science communicators and other commentators of different persuasions. In the context of climate change, this debate has a long tradition. As early as 1996, Stephen Schneider, professor of environmental biology and global change at Stanford University, USA, defended what he called “responsible advocacy” in an article entitled “Is the science/advocate an oxymoron?” 1. He stressed that when scientists adopt an advocacy position, it is important that they are conscious of their values and make those values clear in the context of the position they are advocating. He also pointed out that in science communication, simplification and the use of metaphors is necessary, but that the metaphors chosen should not violate or distort the truth.

The issue of advocacy never went out of fashion in the context of climate change, where science and politics collide on an almost daily basis…

The issue of advocacy never went out of fashion in the context of climate change, where science and politics collide on an almost daily basis, much more so than in synthetic biology. The topic flared up again in 2013 after an article by climate scientist and communicator Tamsin Edwards who argued that climate scientists should not advocate particular policies 2. In contrast, Kevin Anderson, of the Tyndall Centre for Climate Change in the UK, claimed that scientists who remain silent implicitly advocate the status quo. He said in an interview that, “there are no such things as scientists that are not political. Scientists by their nature are being political, whether they engage or do not engage in the wider debates. And I would argue that the ones who are the least political are the ones who engage in it” 3. Silence, in the sense of abstaining from communication, is seen here as a political act, an implicit act of advocacy.

On the same day that Anderson was interviewed (17 December, 2013), a workshop took place at Imperial College London entitled “Silence in the History and Communication of Science”. In the invitation to the workshop, the organizer, Felicity Mellor from Imperial College, London, UK, wrote: “Whilst the public communication of science and public engagement with science are important ideals, there are times when it is expedient and appropriate for scientists to withdraw from the public sphere. The qualities of such withdrawals will vary, from professional silences prompted by competition and fears of plagiarism, to reticence in the face of uncertain knowledge” (http://www.imperial.ac.uk/science-communication-unit/research/silences-of-science/silence-in-the-history-and-communication-of-science/).

These recent debates in the context of climate change reveal some of the dilemmas at the heart of science communication, science advocacy and RRI. Speaking up on a topic can lead to being cast as an (irresponsible) “advocate” or cheerleader, but speaking up is increasingly seen as the responsibility of scientists, in particular by people in high office, such as the UK's Chief Scientific Advisor, Sir Mark Walport (http://www.csap.cam.ac.uk/news/article-mark-walport-csap-lecture-on-climate-change/). This complex relationship between science, communication and responsibility is little understood—and quite easily misunderstood—and needs more research. Every act of communication and every act of silence opens up a space for interpretation and misinterpretation and for power struggles over who should speak for whom, who has the right and responsibility to speak about what, and, of course, when is the right time to speak and when is the right time to remain silent. How scientists and social scientists responsibly use speech and silence to negotiate and advocate common needs still remains a democratic conundrum.

Every act of communication and every act of silence opens up a space for interpretation and misinterpretation and for power struggles…

Jane Calvert and Paul Martin addressed the role of social scientists in raising awareness about synthetic biology in 2009 4. The authors observed that scientists anticipate that synthetic biology research could have the “potential to be extremely contentious” and that at least some scientists accept that regulatory, ethical and social aspects need to be taken into account. However, since the article was published, public awareness of synthetic biology has stayed low, until the advent of “gene editing”—which is rarely linked to synthetic biology. In general, the mass media have been relatively silent on synthetic biology, and debates about ethics and responsibility have remained a matter for a select few rather than society as a whole. Here, we explore some of the reasons for this silence and its implications for RRI.

There is a real paradox then: What are the social and ethical implications of “creating” opinions about synthetic biology, a process that cannot avoid providing information?

Last year, a *Public Attitudes to Science* survey in the UK 5 found that synthetic biology was a topic about which people generally felt least well informed, which has remained the same since 2011. By contrast, people feel much better informed about climate change and vaccination. Interestingly, these topics are associated with polarized attitudes, and, as the survey report points out, it is important to recognize that those people who feel “informed” may not have received scientifically accurate information. With relation to climate change, it was estimated that 13% of people surveyed who felt informed on this issue still believed that human activity does not have a significant impact on the climate. A 2013 Woodrow Wilson Center poll, based on 800 US adults, found that public awareness of synthetic biology and nanotechnology has not changed since previous surveys. 23% of adults said they have heard some or a lot about synthetic biology, compared with 31% who say the same about nanotechnology. Those surveyed mainly associated synthetic biology with being unnatural, artificial and having to do with reproducing life. A poll carried out for the Parliamentary Office of Science and Technology in the UK also found that awareness of synthetic biology was low.

Research in the many fields associated with synthetic biology is, it seems, being carried out and communicated in a context where large parts of the general population are not aware of it, feel they are not well informed about it and have very stereotypical impressions of what it entails. As in the context of climate change, these impressions might lead to polarized attitudes. Here, we focus on this lack of public communication and debate about synthetic biology, in particular the lack of general information in the mainstream press—almost a deadly silence, in fact—and what this might mean for raising awareness responsibly.

Another Woodrow Wilson Center report on *Trends in American and European Press Coverage of Synthetic Biology* 6 found that media coverage of synthetic biology increased significantly between 2008 and 2011. However, US media reports were mainly driven by announcements of the creation of the first synthetic self‐replicating cell by the J. Craig Venter Institute, and the associated *Commission for the Study of Bioethical Issues* investigation instigated by the Obama administration. Even though Craig Venter became a media‐savvy advocate for synthetic biology, framing it as a solution for greening the planet, the report shows that in the US, media coverage was mainly related to issues of biosecurity between 2003 and 2008. In Europe, media reports are mostly focussed on ethical concerns, followed by biosafety and biosecurity matters. This means that in terms of framing, fear dominated over hope.

We investigated these trends in a bit more depth, using the news database LexisNexis to search for synthetic biology in “All English Language News”. We charted developments over the past six years from two years before Craig Venter's major announcement about synthetic biology to the end of 2014. We disaggregated news items according to news sources, such as “Newswires and press releases”, “Newspapers”, “Magazines”, “Industry and Trade press” and so on (Fig 1). “News” about synthetic biology is dominated by “newswires” and “press releases”, which includes news about major synthetic biology businesses. Traditional newspapers only make up a small part of the “news” about synthetic biology, and, although interest since 2010 has not been dwindling as much as we expected, it has also not greatly increased. This seems to indicate that synthetic biology is still not a topic of public interest, while it certainly is one of increasing market interest.

**Figure 1:**
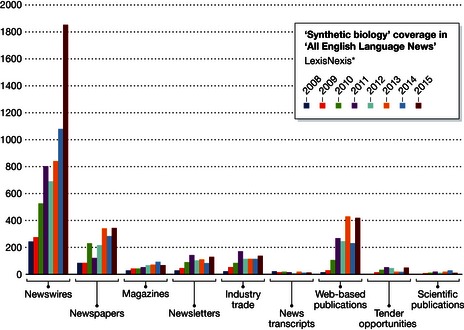
“Synthetic biology” in “All English Language News” (LexisNexis, high similarity setting) Nexis® is a convenient source for the analysis of the traditional print media, although there are some limitations. A significant drawback is that this data source is frequently reconfigured, leading to slightly different results obtained when consulting Nexis® at different dates. Nevertheless, it provides a sufficiently large corpus so as to identify general social representations prevalent at a given point.

As RRI involves assessing commercial and scientific interests together with social, ethical and environmental concerns, how can this be done when there is scarcity of public information and debate about synthetic biology, while at the same time there is surge in industrial research and development? Indeed, when we further investigated some of the commercial and academic press releases, it became clear that engaging with ethical and social issues is generally regarded as an obstacle to commercial growth, rather than integral to responsible research, innovation and commercialization. A 2014 report, *Synthetic Biology Market by Tool—Global Forecast to 2018* claims that, “ethical and social issues such as biosafety and biosecurity are major factors that are restricting the growth of this market” (see Sidebar A). Industry's perception of “ethical and social issues” as obstacles may therefore hamper open and transparent public deliberations about synthetic biology in the context of RRI.

It is time that social science and humanities scholars look more closely at the ethical challenges of science communication and science‐media interactions

Furthermore, only biosafety and biosecurity are mentioned as potential ethical and social issues related to synthetic biology. Ideally, RRI would demand that a much wider array of social and ethical issues are tackled over and above biosafety and biosecurity. These would also have to be specific for the particular research in question and differ substantially between, say, tissue regeneration, biofuels, green chemicals, flavours, fragrances and, above all, gene editing, which is beginning to attract attention in the press, as can be seen in Fig 2. Here, we see a large increase of media coverage in 2015. Again, as with synthetic biology, industry press releases dominate this coverage, while newspapers only started to report on the issue in 2015 and at a much lower volume compared to press releases from industry in particular.

**Figure 2:**
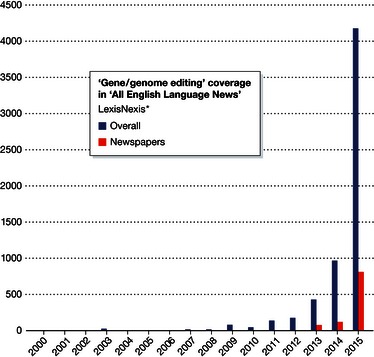
The rise of “gene/genome editing” in “All English Language News” Nexis® is a convenient source for the analysis of the traditional print media, although there are some limitations. A significant drawback is that this data source is frequently reconfigured, leading to slightly different results obtained when consulting Nexis® at different dates. Nevertheless, it provides a sufficiently large corpus so as to identify general social representations prevalent at a given point.

Talking with members of the public about such issues may be difficult for two reasons: first, as we have demonstrated above, the mainstream press remains relatively silent about most of these technologies, so accessing trusted information might be difficult. Second, the market might be growing and diversifying faster than science communication, regulation and responsible innovation efforts around synthetic biology.

Virgil Rerimassie and Dirk Stemerding recently wrote a report for the Dutch Rathenau Institute *Synbio Politics: Bringing synthetic biology into debate* 7 through which they wanted to stimulate “political and societal opinion making” around synthetic biology. They stress that synthetic biology is still in its infancy, that there is a low awareness of the field and that “a broader societal and political debate on synthetic biology has not yet started”. This poses serious problems for science communication and RRI. Practitioners will have to ask: What do we communicate “about” in relation to synthetic biology, what can we write about it and how do we get people to think about and engage with in terms of synthetic biology and RRI? Can one debate an issue or a set of issues in the absence of awareness and information? And what responsibility do social scientists have in this process? Should they stimulate debate (“opinion making”) before it occurs naturally, that is engage in “upstream debating” if you like? Can they do so without risking their neutrality and credibility?

David Rejeski, Director of the Science and Technology Innovation Program at the Wilson Center, argues that, “scientists need to get over trying to tell people what synthetic biology is, and talk about how it is going to be applied and why people should care” 8. This advice seems to be sensible at first glance. However: Applied to what? Are there any applications that “people” are aware of, should be aware of and why? And: Care about what? Before people care about something, they have to be aware of it. Once aware of something people might first ask: What is synthetic biology (all about)? This brings us back to the survey findings, which showed that people felt they were not well informed about synthetic biology. They do feel well informed about climate change though and some people care about this issue quite deeply despite the fact that some of the information they consume may not be scientifically accurate.

There is a real paradox then: What are the social and ethical implications of “creating” opinions about synthetic biology, a process that cannot avoid providing information? The problem is that information is never pure: it is always filtered in some way or will be perceived as such by the audience. This affects the creation of appropriate information or messages and the creation or shaping of public opinion and indeed the creation of “publics”. This also impacts on the perceived neutrality and credibility of those who craft the messages and create publics, that is the scientists, social scientists, journalists or professional intermediaries. The question we as social scientists have to ask ourselves in this context is: Should we engage in “political and societal opinion” making, as advocated by Rerimassie and Stemerding, in the absence of general press coverage and public awareness of, and interest in, a controversial science such as synthetic biology?

So what can we take away from comparing the case study on climate change (responsible advocacy) with the dilemmas of raising awareness about synthetic biology (responsible communication)? One key difference is the role played by the popular media. Climate change has been the focus of enormous media attention during the past decade. This arguably is connected to debates between scientists themselves (at least initially) about humanity's role in climate change, which spilled out into the public and the political sphere. As controversy over climate science has continued, climate scientists have been expected to take some sort of advocacy position, in fact to assume responsibility, rightly or wrongly, for at least advising on policies and their implementation. It is almost impossible for climate scientists to be “neutral” in this highly politicized context and their responses, whether explicit or implicit, are then fed back through media reporting.

In contrast, synthetic biology has, as yet, had very little attention from the popular media. To date, there have been no major disputes between scientists about research or present developments. Some concerns have been raised by citizen or stakeholder organizations 9, but these have not spilled over into the public or political sphere. However, this does not make communicating about synthetic biology any less complex.

It is time that social science and humanities scholars look more closely at the ethical challenges of science communication and science–media interactions, that is to say, at issues related to “responsible language use” or “responsible communication”, as these have policy and politics implications, just as much as “responsible innovation”. Communicating about synthetic biology interferes with a media, policy, information and public awareness ecosystem that is as complex and as delicate as any biological one. Whatever issue science communicators or RRI experts select for “political and societal opinion making” will interfere with this ecosystem in ways that are not easy to anticipate. As Tim Radford, an experienced science writer, once said when reflecting on his own reporting on advances in stem cell research: “The act of writing about something—to choose one topic from the hundred or so potential topics delivered every day in the scientific press—is to hype it. I have chosen this finding rather than that, or the other, so it must be more important, more compelling, more exciting. I select, therefore I hype” 10.

RRI is based on a broad foundation of “engagement” and on (at least) four pillars of “anticipation”, “reflection”, “inclusion” and “responsiveness”. Engagement means “opening up” a broad spectrum of visions, impacts and questions to broader deliberation and debate. We should be aware that selecting which visions and questions we open up for debate, or which words or stories we choose to elicit broader visions, will inevitably sideline, silence or close down others. We need to be prepared to examine the ethical and social implications of such choices for public communication, public awareness, public debate and public understanding of synthetic biology. We argue that an important component in raising awareness responsibly is to engage in dialogues and collaboration between scientists, social scientists and outreach or science communication experts, to help to ensure that there is monitoring of, and reflection on, this process and its impacts.

## Conflict of interest

The authors declare that they have no conflict of interest.

**Sidebar A: Further reading**

**On the aims of Responsible Research and Innovation**

Stilgoe J, Owen R, Macnaghten P (2013) Developing a framework for responsible innovation. *Res. Policy* 42: 1568–1580

von Schomberg R (2013) A vision of responsible innovation. In: *Responsible Innovation: Managing the Responsible Emergence of Science and Innovation in Society*, Owen R, Heintz M, Bessant J (eds). London: John Wiley

**On surveys of public awareness of Synthetic Biology**

Hart Research Group (2013) *Awareness & Impressions of Synthetic Biology*. Wilson Center. Available at: https://www.wilsoncenter.org/publication/awareness-impressions-synthetic-biology

**On Synthetic Biology reports**

POST (2015) *Regulation of Synthetic Biology*. Parliamentary Office of Science and Technology. Available at: http://researchbriefings.files.parliament.uk/documents/POST-PN-0497/POST-PN-0497.pdf

Sutcliffe H (2011) *A Report on Responsible Research and Innovation*. Available at: https://ec.europa.eu/research/science-society/document_library/pdf_06/rri-report-hilary-sutcliffe_en.pdf

**On markets and synthetic biology**

MarketsandMarkets.com (2014) *Synthetic Biology Market by Tool – Global Forecast to 2018*. Available at: http://www.marketsandmarkets.com/Market-Reports/synthetic-biology-market-889.html

Synthetic Biology Project (2015) *Synthetic Biology Products and Applications Inventory*. Wilson Centre. Available at: http://www.synbioproject.org/cpi/
